# Supplementary material for: Photo-Realistic Interactive Virtual Environments for Neurorehabilitation in Mild Cognitive Impairment (NeuroVRehab.PT): A Participatory Design and Proof-of-Concept Study
Source: J Clin Med. 2020 Nov 26;9(12):3821. doi: 10.3390/jcm9123821 (PMC7760013; doi:10.3390/jcm9123821)
Supplement: Supplementary file 1 [file jcm-09-03821-s001.pdf]

Supplementary Files

Table S1. COREQ (COnsolidated criteria for REporting Qualitative research) checklist.

Topic	Item No.	Guide Questions/Description	Reported on Page No.
		**Domain 1: Research team and reflexivity**	
		Personal characteristics	
Interviewer/facilitator	1	Which author/s conducted the interview or focus group?	3 and 7
Credentials	2	What were the researcher's credentials? E.g. PhD, MD	7 and 13
Occupation	3	What was their occupation at the time of the study?	7 and 13
Gender	4	Was the researcher male or female?	7
Experience and training	5	What experience or training did the researcher have?	7
		Relationship with participants	
Relationship established	6	Was a relationship established prior to study commencement?	6
Participant knowledge of the interviewer	7	What did the participants know about the researcher? e.g. personal goals, reasons for doing the research	6
Interviewer characteristics	8	What characteristics were reported about the inter viewer/facilitator? e.g. Bias, assumptions, reasons and interests in the research topic	6 and 13
		Domain 2: Study design	
		Theoretical framework	
Methodological orientation and Theory	9	What methodological orientation was stated to underpin the study? e.g. grounded theory, discourse analysis, ethnography, phenomenology, content analysis	7
		Participant selection	
Sampling	10	How were participants selected? e.g. purposive, convenience, consecutive, snowball	6
Method of approach	11	How were participants approached? e.g. face-to-face, telephone, mail, email	6
Sample size	12	How many participants were in the study?	6
Non-participation	13	How many people refused to participate or dropped out? Reasons?	6
		Setting	
Setting of data collection	14	Where was the data collected? e.g. home, clinic, workplace	7
Presence of non-participants	15	Was anyone else present besides the participants and researchers?	7
Description of sample	16	What are the important characteristics of the sample? e.g. demographic data, date	6 - 7
		Data collection	
Interview guide	17	Were questions, prompts, guides provided by the authors? Was it pilot tested?	Yes, UX included specific questions and prompts. No pilot test was required
Repeat interviews	18	Were repeat inter views carried out? If yes, how many?	No
Audio/visual recording	19	Did the research use audio or visual recording to collect the data?	7 and Doc S1
Field notes	20	Were field notes made during and/or after the interview or focus group?	Yes, field notes were taken
Duration	21	What was the duration of the interviews or focus group?	7
Data saturation	22	Was data saturation discussed?	8
Transcripts returned	23	Were transcripts returned to participants for comment and/or correction?	No
		**Domain 3: analysis and findings**	
		Data analysis	
Number of data coders	24	How many data coders coded the data?	7

Description of the coding tree	25	Did authors provide a description of the coding tree?	8
Derivation of themes	26	Were themes identified in advance or derived from the data?	7
Software	27	What software, if applicable, was used to manage the data?	Not applicable
Participant checking	28	Did participants provide feedback on the findings?	No
		Reporting	
Quotations presented	29	Were participant quotations presented to illustrate the themes/findings? Was each quotation identified? e.g. participant number	8 - 11
Data and findings consistent	30	Was there consistency between the data presented and the findings?	3 - 4; 10 - 12
Clarity of major themes	31	Were major themes clearly presented in the findings?	7 - 11
Clarity of minor themes	32	Is there a description of diverse cases or discussion of minor themes?	7 - 11

*Developed from: Tong A, Sainsbury P, Craig J. Consolidated criteria for reporting qualitative research (COREQ): a 32-item checklist for interviews and focus groups. *International Journal for Quality in Health Care*. 2007. Volume 19, Number 6: pp. 349 – 357

Doc S1. Ethical standards

Participants recruitment

All participants (phase 1 and 3) were informed of the scope and objectives of the study and their right to withdraw from the study at any moment. An informative sheet with the study's aims, procedures and researchers' contacts was provided to all participants. Written informed consent was gathered just after participants' questions were clarified.

In phase 1 of this project, it was explained to older adults that no information concerning the supermarket chain they usually go, the amount of money spent, or brands purchased would be collected. In phase 3, health professionals were informed that interviews would be audio-recorded, and in addition to written informed consent, verbal permission to start audio-recording the session was asked before the beginning of the interviews.

Supermarket image capture

A local Portuguese supermarket was contacted and asked for permission to capture images of the inside of the store. Image capture was performed according to The Right to Information provided in the General Data Protection Regulation, published on 4 May 2016. A sign, warning customers about the image capture was displayed at the store entrance and at the aisles near to where the photographs were being taken. Image capture was done during low affluence periods (three consecutive morning periods), and capture of supermarket users' silhouette (*i.e.*, customers and employees) was avoided.

After image collection, an editing process took place to ensure the anonymity, protection, and respect for the identity and property of all parties involved. Supermarket users' silhouettes that were unintentionally captured, as well as store security system elements (*i.e.*, video surveillance cameras), were removed (not blurred). All brands were blurred. Image editing was done by the first author (FFB), and except for supermarket's logo removal and brand blur, which were done posteriorly, other research team members only had access to the edited images.

Table S2. Shopping Behaviors Questionnaire with Older Adults descriptive data (*n* = 110).

**Demographic data**		
**Age (mean ± SD; range)**	**70.92 ± 5.94 (61 – 86)**	
Sex (*n*, %)	Male: 28 (26.7%)	Female: 77 (73.3%)
Professional Status (*n*, %)	Retired: 93 (96.9%)	On active: 3 (3.1%)
Questionnaire items*	Multiple choice answers	*n* (%)
1. Usually I shop at …	Local grocery store	8 (8.1%)
	Supermarket	53 (53.5%)
	Hypermarket or department store	38 (38.4%)
2. Usually I go shopping …	By myself	64 (60.4%)
	With my husband/wife	37 (34.9%)
	Family member	3 (2.8%)
	Friend	1 (0.9%)
	Other	1 (0.9%)
3. Usually I make my shopping list before going shopping?	Yes	59 (56.2%)
	No	43 (41.0%)
	No, I use another strategy	3 (2.9%)
4. I organize my shopping list by …	Alphabetic order of the name of the products	1 (1.5%)
	The order I will visit the different supermarket sections	10 (14.7%)
	As I remember what I need to buy	54 (79.4%)
	Use another strategy	3 (4.4%)
5. I use my shopping list (or another strategy) while I am shopping?	Yes	42 (61.8%)
	Sometimes	16 (23.5%)
	No	10 (14.7%)
6. Usually, when I go shopping …	I shop for the entire month	21 (20.0%)
	I shop for the week	59 (56.2%)
	I shop thinking of specific dishes	17 (16.2%)
	I shop considering the next meal I will have	8 (7.6%)
7. Usually, I go shopping …	Less than once per week	56 (52.8%)
	2 – 3 times per week	39 (36.8%)
	4 or more times per week	11 (10.4%)
8. On average, I spend (in minutes) shopping. (mean ± SD; range, mode)	59.24 ± 33.68 minutes [10 – 180]<br>Mode = 60 minutes	
9. When I cannot find a product at the supermarket, I …	I ask for help to the employees	67 (62.0%)
	Try to find the signs that identified the various supermarket sections	29 (26.9%)
	Go through the whole supermarket until I find what I'm looking for	12 (11.1%)
10. Before I pay, I …	Know exactly how much I'm going to pay.	7 (6.7%)
	Know more or less how much I will spend	61 (58.1%)

		Know how much I will spend if I don't buy more than 3 products	n/a
		Don't know and wait the cashier tell me	37 (35.2%)
11.	When I pay in cash …	I know exactly how much money I have to receive in return.	33 (35.9%)
		I know more or less how much money I have to receive in return.	16 (17.4%)
		I only check if the change is correct.	30 (32.6%)
		I do not check the change and trust the cashier.	13 (14.1%)

* The following items are a free translation from Portuguese to English of items from the Shopping Behaviors Questionnaire with Older Adults.
